# Shyness and Learning Adjustment in Senior High School Students: Mediating Roles of Goal Orientation and Academic Help Seeking

**DOI:** 10.3389/fpsyg.2018.01757

**Published:** 2018-09-19

**Authors:** Yingmin Chen, Liang Li, Xiaoyi Wang, Yingli Li, Fengqiang Gao

**Affiliations:** ^1^School of Psychology, Shandong Normal University, Jinan, China; ^2^Weifang First Middle School, Weifang, China

**Keywords:** shyness, goal orientation, academic help seeking, learning adjustment, senior high school student

## Abstract

Learning maladjustment is a common phenomenon in the context of examination-oriented education system in china, especially among high school students who experience intense pressure when preparing for the national college entrance examination. Previous literature suggests that shyness may negatively affect ones’ cognition, emotion, and behavioral performance and lead to academic and social maladjustment. Therefore, learning adjustment among shy high school students is a critical and practical point of inquiry. With a sample of 677 Chinese senior high school students, this study aims to assess the association between shyness and learning adjustment and related mechanisms of goal orientation (i.e., mastery-approach goals, mastery-avoid goals, performance-approach goals, and performance-avoid goals) and academic help seeking (i.e., instrumental help seeking from teacher, instrumental help seeking from classmate, executive help seeking, and avoidance of help seeking). Self-report measures were adopted to collect information on: demographic characteristics, the level of shyness, goal orientation, academic help seeking, and learning adjustment. Results indicated that shyness was negatively correlated with learning adjustment, and this association was mediated by the dimensions of goal orientation and dimensions of academic help seeking. Specifically, shyness not only predicted learning adjustment through mastery-approach goals, and instrumental help seeking (teachers) but also predicted learning adjustment through the multiple mediating effects of the dimensions of goal orientation and the dimensions of academic help seeking (i.e., mastery-approach goals and instrumental help seeking from teachers, mastery-approach goals and executive help seeking, mastery-avoid goals and instrumental help seeking from classmates, mastery-avoid goals and executive help seeking, and performance-avoid goals and executive help seeking). Identifying these mediators further enables us to work out effective measures to promote shy high school students’ learning adjustment.

## Introduction

Senior high school education is a key stage laying a solid foundation for students’ lifelong development. High school students are going to face many adaptative and developmental tasks in this stage, while adaptation and development in learning field is one of the most important development tasks (Deb et al., 2015). The extent to which adolescents succeed in learning adjustment critically influences their academic achievement, peer relationship, and even subsequent educational opportunities and choices (Farmer et al., 2009; Ryan, 2011), which in turn might facilitate adolescents’ adjustment and mental well-being in this period of life.

Learning adjustment refers to the process through which students make efforts to achieve balance in their learning environment and improve their academic performance (Nie et al., 2004), consisting of learning habits, utilization of learning resources, learning motivations, learning satisfactions, learning styles, etc. In the context of examination-oriented education systems, learning maladjustment is a common phenomenon, especially among Chinese high school students who experience intense pressure when preparing for the national college entrance examination (Kuperminc et al., 2008; Liu and Chen, 2010; Yüksel, 2016). Learning maladjustment may adversely affect Chinese high school students’ aspects of school adaptation as well as their future development (Song, 2015; Longobardi et al., 2016).

### Shyness and Learning Adjustment

Reciprocal interaction theory (Bandura, 1976) stressed the influence of a reciprocal interaction between environmental, personal, and behavioral factors on individual developments, with particular emphasis on the influence of personal factors. According to Bronfenbrenner (1979), individual factors refer to individuals characteristics (gender, race, age, experience, personality, etc.); environmental factors refer to the environmental characteristics of individual survival and development, including macro-environment (socio-cultural background, socio-economic development, etc.) and micro-environment (family, teachers, peers, etc.). In the literature, identified factors that affect learning adjustment among high school students include environmental factors (e.g., family socioeconomic status, parenting style, teacher support, peer friendship, and social environment) (Hair and Graziano, 2003; Verner-Filion and Gaudreau, 2010; Butler, 2011; Garg et al., 2016) and individual factors (e.g., personality, intelligence, achievement motivation, and academic self-efficacy) (Powers et al., 2005; Gunnoe, 2013; Shin and Ryan, 2014; Larose et al., 2018). In all personal variables, researchers have found that shyness as a personality trait affected individuals’ learning adjustment (Chen et al., 1995; Liu et al., 2012; Yang et al., 2015; Coplan et al., 2017). Shyness refers to behavioral responses such as inhibition and withdrawal in response to social and novel situations. Shyness can stem from fear of negative evaluation and may be accompanied by emotional distress or inhibition; thus, shyness can interfere with desired participation in activities and the pursuit of personal and professional goals (Henderson and Zimbardo, 2001). High school is a key period in social and personality development among teenagers, whose internalization of difficulties (e.g., shyness, social anxiety, and depression) is especially serious (Hu et al., 2015). Intense shyness may negatively affect an individual’s cognition, emotions, and behavioral performance and lead to learning and social maladjustment (Liu et al., 2012, 2014, 2015; Ponti and Tani, 2015). Moreover, previous studies have found shyness predicted poor learning adjustment among junior high school students (Chen et al., 2017; Coplan et al., 2017).

Although shyness has been associated with learning maladjustment in considerable research conducted in China (Chen et al., 1995, 2011; Yang et al., 2015; Coplan et al., 2017), to date insufficient interest has been devoted to studying the mechanisms that underlie this relation. Moreover, most studies on shyness and learning adjustment have focused on children; so far, only a few studies have investigated related mechanisms (e.g., self-esteem, coping style, and teacher–student relationship) on the association between shyness and learning adjustment in high school students (Feng et al., 2014; Wu, 2015; Chen et al., 2017); thus, findings are of limited significance. Therefore, the mechanism of the effect of shyness on learning adjustment among high school students warrants investigation. In this study, multiple mediation analysis was adopted to investigate the mechanisms through which shyness leads to learning maladjustment.

### Shyness, Academic Help Seeking, and Learning Adjustment

Academic help seeking is a two-part process that supports successful learning outcomes (Karabenick and Newman, 2006; Barnard et al., 2008). First, students must recognize the need for help, and then must decide whether to actually request help (Ryan and Pintrich, 1997). According to Karabenick and Newman (2006), academic help seeking include instrumental help seeking from teacher (students ask for the help needed from teacher in order to learn independently), instrumental help seeking from classmate (students ask for the help needed from classmates in order to learn independently), executive help seeking (students attempt to avoid work by asking others for answers to problems), and avoidance of help seeking (students would rather write an answer than asking others for the answer to the problem). Previous research has reported that academic help seeking explains the internal mechanism of students’ learning adjustment. When students solve their academic problems by asking for help from others, thereby enhancing their understanding, they exhibit adaptive academic behavior (Chen et al., 2018).

Holt (2014) argued that academic help seeking mediates the association between parent attachment and learning adjustment; close parent–child relationships are associated with less shame and embarrassment on the part of the child with respect to obtaining academic assistance; this attitude predicts other positive academic behavior, including organization, preparation, and classroom engagement. However, highly shy students who are extremely concerned about how others view them tend to restrain their behavior and may not communicate effectively with others. When they ask help from others, they may feel more shameful and embarrassed. These personality characteristics may inhibit a shy individual from seeking help from others (such individuals may engage in negative help-seeking methods or avoid help seeking altogether) upon encountering academic difficulties; consequently, problems may not be solved promptly or effectively, thereby leading to the student in question experiencing learning maladjustment. Yap et al. (2013) argued that embarrassment or shyness was the most frequently occurring barrier to seeking help among people aged 15–25 years. Shy individuals seek help less frequently (or avoid seeking help) and take substantially more time to seek help than those with low shyness with respect to task completion challenges (Horsch, 2006). Another line of research suggested that shyness may affect individuals’ academic help-seeking behavior, higher levels of shyness corresponded with more passive academic help-seeking behavior (executive help seeking, avoidance of help seeking), whereas lower levels of shyness corresponded with active academic help-seeking behavior (instrumental help seeking) (Dou et al., 2015). As a consequence, highly shy students are prone to adopt passive academic help-seeking behavior rather than active academic help-seeking behavior, thereby resulting in learning maladjustment. These findings suggest that the negative association between shyness and learning adjustment can be partly accounted for by academic help seeking.

### Shyness, Goal Orientation, and Learning Adjustment

Goal orientation refers to an individual’s plan for processes that determine cognitive, emotional, and behavioral outcomes (Feltman and Elliot, 2012; Eder et al., 2013). In the influential individual-level 2 × 2 framework (Elliot and McGregor, 2001), four distinct types of achievement goals are identified: mastery approach goals (focused on developing competence and learning new things), mastery avoid goals (focused on avoiding incompetence relative to absolute or interpersonal standards), performance-approach goals (focused on demonstrating competence), and performance-avoid goals (focused on avoiding demonstrations of incompetence). Mastery goals (mastery-approach and mastery-avoid goals) correspond with incremental views of ability; individuals believe that they can improve their abilities by mastering knowledge. By contrast, performance goals (performance-approach and performance-avoid goals) correspond to entity-based views of ability, namely, the belief that people’s abilities are fixed and they may complete tasks merely to prove their abilities (Elliot and McGregor, 2001; Eder et al., 2013). Studies have demonstrated that achievement goal orientation can influence students’ learning adjustment (Verner-Filion and Gaudreau, 2010; Tuominen-Soini et al., 2012). High-performance goals and low mastery goals may lead to poor academic satisfaction (Verner-Filion and Gaudreau, 2010); mastery-oriented students were highly engaged in their studies and perceived their schoolwork as meaningful (Tuominen-Soini et al., 2012).

According to achievement goal theory (Elliot and Thrash, 2002), individuals with a higher propensity toward mastery goals often compare their current state with that of their previous selves. Such individuals repeatedly perform difficult tasks to improve their abilities (mastery approach) or strive to accomplish tasks and master knowledge (mastery avoid); their progress and mastery of knowledge serve as motivators (Elliot and Thrash, 2002; Mierlo, 2015). By contrast, individuals with a higher propensity toward performance goals often compare themselves with others. Such individuals strive to exhibit performance superior to others (performance approach) or avoid performing in a manner inferior to others (performance avoid), and are motivated by positive external feedback (Elliot and Thrash, 2002; Mierlo, 2015; Sharma and Nasa, 2016). This feedback-based motivation is similar to the fear of negative evaluation (Henderson and Zimbardo, 2001). Empirical studies have explored the association between shyness and goal orientation. For example, Fallah (2014) found that the willingness of shy students to communicate in English was mediated by their motivation, perhaps one way to increase shy students’ willingness to communicate in English is to enhance their motivation. Another study explored the relation between shyness and language learning motivation (Mohammadian, 2013), and found language learning motivation mediated the relation between shyness and language learning tasks; this study has further shown that individuals with a high level of task orientation (e.g., mastery-approach goals and mastery-avoid goals) use more active cognitive strategies and self-constraints in their learning; however, individual with a high level of ego orientation (e.g., performance-approach goals and performance-avoid goals) use more motivational strategies, that is, to surpass others and avoid being negatively evaluated by others (consistent with shyness). Therefore, in order to prevent future possible failures and negative evaluations, these individuals continue to try to use these motivational strategies (Elliot et al., 1999; Riveiro, 2001). Based on these findings, this study posits that higher levels of shyness may have higher performance goals and lower mastery goals, resulting in learning maladjustment, that is, the negative effect of shyness on learning adjustment may be partly mediated by goal orientation.

### Goal Orientation and Academic Help Seeking

Numerous studies have indicated that differences in goal orientation influence students’ different academic help-seeking behavior. For example, Nosaki (2003) found that mastery goal orientation indirectly influenced adaptive help seeking because the observed students felt useless. Conversely, performance goal orientation indirectly influenced dependent help seeking and avoidance of help seeking because the observed students felt uncertain or inferior with respect to their competence. Additional studies have reported that task-based goals (mastery goals) are positively associated with adaptive help-seeking behavior (instrumental help seeking) and negatively associated with maladaptive help-seeking behavior (executive help seeking and avoidance of help seeking). By contrast, performance-avoid goals are positively associated with maladaptive help seeking and negatively associated with adaptive help seeking (Oberman, 2002; Cheong et al., 2004). In addition, Newman (2002) indicated that adaptive help seeking (e.g., instrumental help seeking) is a strategy based on self-regulated learning. Specifically, when independently engaged in difficult tasks, students require initial motivation to assess their status and situation and must realize that a problem can be solved before initiating help-seeking behavior. Based on the above research, this study posits that students with higher performance goals (performance-approach goals and performance-avoid goals) and lower mastery goals (mastery-approach goals and mastery-avoid goals) may have higher maladaptive help-seeking behavior (executive help seeking and avoidance of help seeking) and lower adaptive help-seeking behavior (instrumental help seeking from teacher or classmate). In view of the association between shyness, goal orientation, academic help seeking, and learning adjustment that has been deduced, this study attempts to explore the multiple mediating role of goal orientation and academic help seeking in the association between shyness and learning adjustment.

### The Present Study

In the context of examination-oriented education systems, learning maladjustment is a common phenomenon, especially among Chinese high school students who experience intense pressure when preparing for the national college entrance examination (Kuperminc et al., 2008; Liu and Chen, 2010; Yüksel, 2016). Shyness is an unfavorable factor in individual socialization (Joiner, 1997; Besic et al., 2009). Intense shyness may negatively affect an individual’s cognition, emotions, and behavioral performance and lead to learning and social maladjustment (Liu et al., 2012, 2014, 2015; Ponti and Tani, 2015). Therefore, learning adjustment among shy high school students is a critical and practical point of inquiry. To enhance the literature, this study investigated the influence of goal orientation and academic help seeking (independent contribution and concurrent contribution) on the association between shyness and learning adjustment in a sample of Chinese high schools students.

**FIGURE 1:**
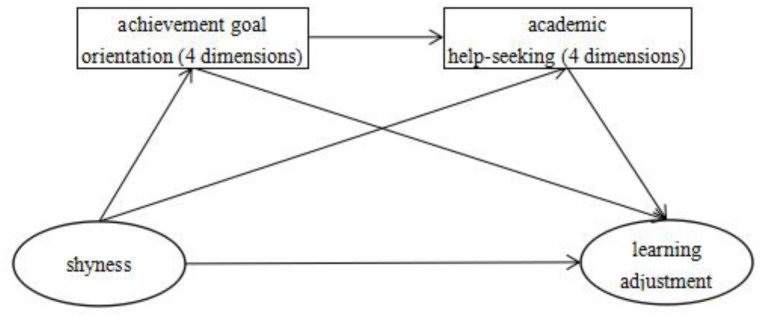
The conceptual model of this study.

Based on the literature review, the following hypotheses were proposed:

H1. Shyness negatively predicts learning adjustment.
H2. The negative effect of shyness on learning adjustment may be mediated by the dimensions of goal orientation (i.e., mastery-approach goals, mastery-avoid goals, performance-approach goals, and performance-avoid goals).
H3. The negative effect of shyness on learning adjustment may be mediated by the dimensions of academic help seeking (i.e., instrumental help seeking toward teachers or classmates, executive help seeking, and avoidance of help seeking).
H4. Shyness predicts learning adjustment through the multiple mediating roles of the dimensions of goal orientation and dimensions of academic help seeking (see **Figure 1**).

## Materials and Methods

### Ethics Statement

This study was carried out in accordance with the recommendations of Ethics Committee of Shandong Normal University, Shandong, China. The protocol was approved by the Ethics Committee of Shandong Normal University. Permission for this study was obtained from parents, school authorities, and principals, and all participants and their parents/legal guardians had signed the informed consent in accordance with the Declaration of Helsinki.

### Participants and Procedure

This study was randomly selected participants from two public secondary schools in Weifang in Shandong province in Mainland China. The participants completed the corresponding questionnaires in the classroom in groups of 20–25 students, on a regular school day, in the presence of an experienced research assistant, and the survey lasted for about 30 min. Finally, 700 students returned the questionnaires and 23 students were excluded because their questionnaires answered regularly or had one or more scales unfinished. Therefore, this study got a final sample of 677 (10th to 12th grade). The participants ranged in age from 15 to 20, 220 were in grade 1, 230 were in grade 2, and 227 were in grade 3. Gender Distribution was 45.20% males and 54.80% females.

### Measure

#### Shyness

The 31-item Shyness Scale for Chinese Middle School Student (SS-CMSS; Chen, 2013) was used to assess the level of shyness of middle school students. This scale, consisting of self-expression shyness (seven items), shyness toward novelty (six items), shyness for negative social evaluation (six items), shyness toward the opposite sex (seven items), unassuming shyness (five items), is scored on a 5-point scale (1 = totally disagree to 5 = totally agree). Sample items include, “I usually hide in a corner when I take part in group activities (self-expression shyness),” “I feel nervous and embarrassed when I stay with someone I don’t know (shyness toward novelty),” “I feel awkward and uncomfortable even with a little criticism from the teacher (shyness for negative social evaluation),” “I blush and feel embarrassed when the opposite sex focus on me (shyness toward the opposite sex),” and “I do things more low-key, I don’t like to be in the limelight (unassuming shyness).” In this study, Cronbach’s alphas for five dimensions ranged from 0.60 to 0.87, and 0.92 for the whole scale. The fit indices from a confirmatory factor analysis were adequate, χ^2^/*df* = 2.698, GFI = 0.905, TLI = 0.909, and RMSEA = 0.050.

#### Goal Orientation

The 29-item Four Point Goal Orientation Scale (FPAGOS; Liu, 2003) was used to assess the level of goal orientation of high school students. This scale, consisting of mastery approach goal (nine items), mastery-avoid goal (five items), performance approach goal (nine items), and performance avoid goal (six items), is scored on a 5-point scale (1 = totally disagree to 5 = totally agree). Sample items include, “I like learning because it allows me to increase my knowledge (mastery approach goal),” “I often worry about not mastering the knowledge taught by the teacher in class (mastery avoid goal),” “I am very happy when others envy me for my grades (performance approach goal),” and “When speaking freely in class, I often worry that my views are childish and dare not speak (performance avoid goal).” In this study, Cronbach’s alphas for five dimensions ranged from 0.79 to 0.84, and 0.86 for the whole scale. The fit indices from a confirmatory factor analysis were adequate, χ^2^/*df* = 2.495, GFI = 0.919, TLI = 0.907, and RMSEA = 0.047.

#### Academic Help Seeking

The 19-item Academic Help Seeking Behavior Scale in Chinese version (AHBS_C; Li and Zhang, 1999) was used to assess the level of academic help seeking behavior. This scale, consisting of instrumental help seeking from teacher (five items), instrumental help seeking from classmate (five items), executive help seeking (four items), and avoidance of help seeking (five items), is scored on a 5-point scale (1 = totally disagree to 5 = totally agree). Sample items include, “I ask the teacher for advice when I do not understand the meaning of a mathematical problem (instrumental help seeking from teacher),” “I ask my classmates for advice when I do not understand the meaning of a mathematical problem (instrumental help seeking from classmate),” “I copy someone else’s answer when I can’t solve a mathematical problem (executive help seeking),” and “I would rather write an answer than ask a teacher or classmate when I can’t solve a mathematical problem (avoidance of help seeking).” In this study, Cronbach’s alphas for four dimensions ranged from 0.65 to 0.87, and 0.70 for the whole scale. The fit indices from a confirmatory factor analysis were adequate, χ^2^/*df* = 3.157, GFI = 0.933, TLI = 0.927, and RMSEA = 0.056.

#### Learning Adjustment Behavior

The 30-item Learning Adaptive Behavior Scale in Chinese version of Social Adaptive Behavior Scale (SABS_C; Nie, 2005) was used to assess the level of learning adjustment behavior. This scale, consisting of learning habits (six items), utilization of learning resources (five items), learning motivations (six items), learning satisfactions (six items), and learning styles (seven items), is scored on a 2-point scale (1 = totally disagree to 2 = totally agree), there is one reverse scoring question in this sub-scale, with higher score indicating higher learning adjustment behavior. Sample items include, “I think positively about the problems in study (learning habits),” “I like to think independently and finish learning tasks (learning styles),” “I use learning reference books (such as dictionaries, reference material) (utilization of learning resources),” “I think the purpose of study is to get a good university and a good job in the future (learning motivations),” and “I am happy when I study (learning satisfactions).” In this study, Cronbach’s alphas for five dimensions ranged from 0.59 to 0.70, and 0.69 for the whole scale. The fit indices from a confirmatory factor analysis were adequate, χ^2^/*df* = 2.552, GFI = 0.946, TLI = 0.907, and RMSEA = 0.039.

## Results

### Common-Method Bias

Since the data were collected in a single instrument from a single respondent from each organization, the threat of common-method bias to the validity of the data was checked using Izenman et al. (1978) one-factor test. The resulting principal component analysis returned 28 distinct factors with eigenvalues greater than 1, which accounted for 59.90% of the variance; the first factor accounted for only 11.46% of the variance. Results indicated that common-method bias was not contaminating the associations between research variables.

### Descriptive Statistics and Correlation Analysis

**Appendix Table A1** summarizes the Pearson bivariate correlations among the variables in the structural models, as well as the mean and standard deviation of each measure. Shyness was significantly related to learning adjustment.

Mastery-approach, mastery-avoid, and performance-approach goals were significantly related to learning adjustment (*p* < 0.01 for all). No significant correlation was observed between performance-avoid goals and learning adjustment (*p* > 0.05). Instrumental help seeking (teachers), instrumental help seeking (classmates), executive help seeking, and avoidance of help seeking were significantly related to learning adjustment (*p* < 0.01 for all).

Mastery-approach, mastery-avoid, and performance-avoid goals were significantly related to shyness (*p* < 0.01 for all); however, no significant correlation was observed between performance-approach goals and shyness (*p* > 0.05). Instrumental help seeking (teachers), executive help seeking, and avoidance of help seeking were significantly related to shyness (*p* < 0.01 for all); however, no significant correlation was observed between instrumental help seeking (classmates) and shyness (*p* > 0.05).

In addition, this study examined gender and grade difference in research variables. Results showed that there was no significant grade difference in any variables in this study. Furthermore, independent sample *t*-tests indicated that there were significant gender difference in instrumental help seeking from classmates, *t*(667) = -4.875, *p* < 0.001, revealing that boys reported lower levels of instrumental help seeking from classmates, *M* (*SD*) = 3.86 (0.80), compared to girls, *M* (*SD*) = 4.13 (0.66), but no significant gender difference in other variables.

### Mediation Analyses

**FIGURE 2:**
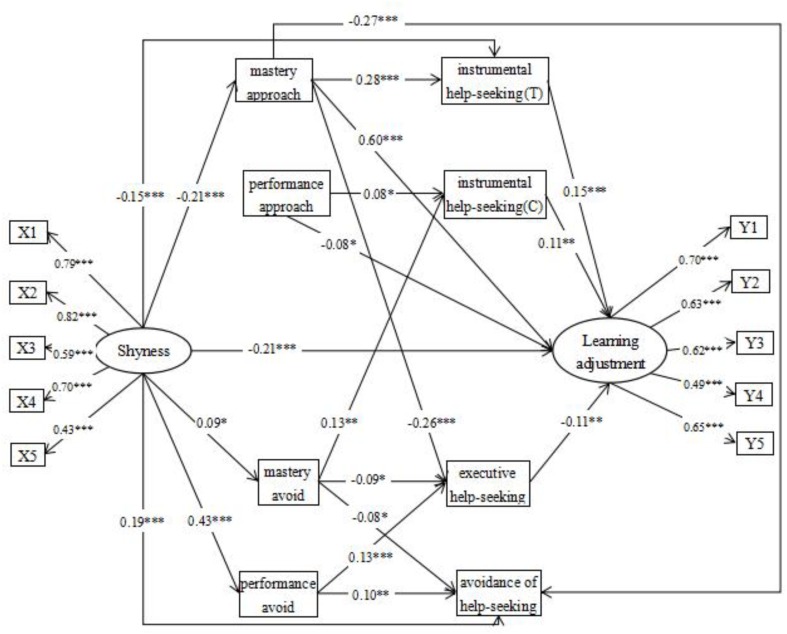
Standardized coefficients in the SEM. X1, self-expression shyness; X2, shyness toward novelty; X3, shyness for negative social evaluation; X4, shyness toward the opposite sex; X5, unassuming shyness; Y1, learning habits; Y2, utilization of learning resources; Y3, learning motivations; Y4, learning satisfactions; Y5, learning styles; instrumental help-seeking (T); instrumental help-seeking from teacher; instrumental help-seeking (C); instrumental help-seeking from classmate. ^∗^*p* < 0.05, ^∗∗^*p* < 0.01, ^∗∗∗^*p* < 0.001.

AMOS 20.0 was used for a multiple mediation analysis (Preacher and Hayes, 2008). Structural equation models were established to examine shyness as a predictive factor in the levels of influence of the dimensions of achievement goal orientation and academic help seeking on learning adjustment. According to procedures for testing for mediating effects (Wen et al., 2006), we applied an SEM approach to assess the following three models: (a) a direct effect model (model 1) with paths from shyness to learning adjustment; (b) an indirect effect model (model 2), for which we used the initial direct effect model as a basis and added mediators (e.g., dimensions of achievement goal orientation and those of academic help seeking) between shyness and learning adjustment, as well as paths from dimensions of achievement goal orientation to those of academic help seeking; and (c) from the multiple indirect effects model, we deleted distinct paths according to the principle of standardized path coefficient ranking from small to large, resulting in model 3. Besides, following the correlation results, we controlled gender based on model 3, resulting in model 4 (see **Figure 2**). As shown in **Appendix Table A2**, the four model fit indices demonstrate that all models fit the data.

As shown in **Figure 2**, shyness not only directly and negatively predicted learning adjustment (β = -0.21, *p* < 0.001) but also indirectly predicted learning adjustment through mastery-approach goals and instrumental help seeking (teachers). Moreover, shyness indirectly predicted learning adjustment through chain mediation of the dimensions of goal orientation and those of academic help seeking through the following five paths. (1) Shyness significantly negatively predicted mastery-approach goals (β = -0.21, *p* < 0.001), mastery-approach goals significantly positively predicted instrumental help seeking (teachers) (β = 0.28, *p* < 0.001), and instrumental help seeking (teachers) significantly positively predicted learning adjustment (β = 0.15, *p* < 0.001). (2) Shyness significantly negatively predicted mastery-approach goals (β = -0.21, *p* < 0.001), mastery-approach goals significantly negatively predicted executive help seeking (β = -0.26, *p* < 0.001), and executive help seeking significantly negatively predicted learning adjustment (β = -0.11, *p* < 0.01). (3) Shyness significantly positively predicted mastery-avoid goals (β = 0.09, *p* < 0.05), mastery-avoid goals significantly positively predicted instrumental help seeking (classmates) (β = 0.13, *p* < 0.01), and instrumental help seeking (classmates) significantly positively predicted learning adjustment (β = 0.11, *p* < 0.01). (4) Shyness significantly positively predicted mastery-avoid goals (β = 0.09, *p* < 0.05), mastery-avoid goals significantly negatively predicted executive help seeking (β = -0.09, *p* < 0.05), and executive help seeking significantly negatively predicted learning adjustment (β = -0.11, *p* < 0.01). (5) Shyness significantly positively predicted performance-avoid goals (β = 0.43, *p* < 0.001), performance-avoid goals significantly positively predicted executive help seeking (β = 0.13, *p* < 0.001), and executive help seeking significantly negatively predicted learning adjustment (β = -0.11, *p* < 0.01).

This study used PROCESS (Hayes, 2012) to test the statistical significance of those indirect effects. In this study, we used 5000 resamples in order to estimate 95% confidence intervals (CIs) (MacKinnon et al., 2007; Hayes and Preacher, 2013). If the 95% CI did not include zero, it meant a statistical significant indirect effect, gender was included as a covariate. As shown in **Appendix Table A3**, a bootstrapping analysis indicated that the indirect effects on shyness on learning adjustment were statistical significant.

## Discussion

For high school students, especially shy students, learning adjustment is crucial and may affect other adaptive processes in school (Hussain et al., 2008). Therefore, this study aims to investigate factors affecting the learning maladjustment of shy high school students. When these factors are identified, effective measures are further expected to be employed to encourage shy high school students to engage in more learning adjustment behavior and further reduce the emergence of internal and external problems, which may promote their mental health and well-being (Burns et al., 2011; Lai et al., 2015).

In this study, a multiple mediation model was used to examine the mechanism of the effect of shyness on learning adjustment. The results indicated that the dimensions of goal orientation and dimensions of academic help seeking played mediating roles in the relation between shyness and learning adjustment. These findings were helpful for educators to put effective measures to reduce the negative influence of shyness on learning adjustment. Integrating this findings and previous studies (Chen et al., 1995; Liu et al., 2012; Shim and Finch, 2014; Yang et al., 2015), one can opt to influence both directly and indirectly the way shy high school students relate to learning maladjustment topics in an attempt to improve their school adjustment. In this way, high school students, especially shy students, could get through their senior high school stage more smoothly and actively adapt to their school life.

### The Association Between Shyness and Learning Adjustment

First, this study found that shyness was negatively associated with learning adjustment, and learning adjustment was negatively predicted by shyness (β = -0.21, *p* < 0.001). This finding suggested that high-shy students were more likely to have problems with learning adjustment; which was consistent with those of previous studies (e.g., Liu et al., 2012; Yang et al., 2015; Coplan et al., 2017) and supported H1. This finding highlighted the negative impacts of shyness as an important personality variable on learning adjustment of high school students. It should arouse the attention of educators to shy high school students’ learning adjustment.

Moreover, this study found that shyness not only directly predicted learning adjustment but also indirectly predicted learning adjustment through goal orientation and academic help seeking, that is, the dimensions of goal orientation and dimensions of academic help seeking played mediating roles between shyness and learning adjustment. Specifically, the dimensions of goal orientation and dimensions of academic help seeking played the multiple mediating roles between shyness and learning adjustment.

### Shyness Predicted Learning Adjustment Through Mastery-Approach Goals and Instrumental Help Seeking (Teachers)

This study found that learning adjustment was indirectly predicted by shyness through mastery-approach goals and instrumental help seeking (teachers). Specifically, intense shyness may have limited individuals’ mastery-approach goals and instrumental help seeking from teachers, resulting in learning maladjustment. Thus, H2 and H3 were partially supported. Shy individuals generally strive to avoid making mistakes and performing tasks poorly, and exert little effort toward acquiring new knowledge or improving their abilities by engaging in challenging tasks; these personality traits can result in learning maladjustment. The fragile theory of academic help seeking suggests that individuals with low self-esteem tend to perceive themselves negatively and are vulnerable when faced with threatening information (Gall, 1985) such as others’ negative evaluations of their abilities, which may occur as a result of requests for help. Therefore, individuals with low self-esteem do not often turn to others for help. Shy individuals typically experience low self-esteem and intense concern regarding others’ evaluations of them (Cheek and Melchior, 1990; Zhao et al., 2012, 2013). This personality trait leads them to regard seeking help from others as a threat to their own ability upon encountering difficulties in their schoolwork. Therefore, shy individuals seldom regard seeking help from others as a primary problem-solving method. Any subsequent failure to solve difficult problems independently may result in poor learning adjustment.

These findings highlighted the influence of mastery-approach goals and instrumental help seeking (teachers) on learning adjustment of shy high school students. These findings suggested that shy individuals’ correct learning motivation can be cultivated; educators can guide students to improve their abilities by mastering knowledge. Furthermore, when students, especially shy students, asked for help, educators can adopt a supportive and encouraging attitude rather than impatient or ridiculous in order to encourage students form good academic help-seeking habits.

### Shyness Predicted Learning Adjustment Through the Multiple Mediating Effects of the Dimensions of Goal Orientation and the Dimensions of Academic Help Seeking

This study found that shyness indirectly influenced learning adjustment through the influence of mastery-approach goals and instrumental help seeking (teachers) (β = -0.21, *p* < 0.001, β = 0.28, *p* < 0.001, β = 0.15, *p* < 0.001, respectively), mastery-approach goals and executive help seeking (β = -0.21, *p* < 0.001, β = -0.26, *p* < 0.001, β = -0.11, *p* < 0.01, respectively), mastery-avoid goals and instrumental help seeking (classmates) (β = 0.09, *p* < 0.05, β = 0.13, *p* < 0.01, β = 0.11, *p* < 0.001, respectively), mastery-avoid goals and executive help seeking (β = 0.09, *p* < 0.05, β = -0.09, *p* < 0.05, β = -0.11, *p* < 0.01, respectively), and performance-avoid goals and executive help seeking (β = 0.43, *p* < 0.001, β = 0.13, *p* < 0.001, β = -0.11, *p* < 0.01, respectively). Thus, H4 was partially supported.

In agreement with previous studies, this study revealed critical links between goal orientation and academic help seeking (Oberman, 2002; Nosaki, 2003; Cheong et al., 2004; Bartels et al., 2011; Okada et al., 2012). Specifically, mastery goals were positively associated with adaptive help-seeking behavior and negatively associated with maladaptive help-seeking behavior. Conversely, performance-avoid goals were positively associated with maladaptive help-seeking behavior and negatively associated with adaptive help-seeking behavior.

The effect sizes of the path through the influence of performance-avoid goals and executive help seeking was the biggest. Shy individuals tend to prioritize task accomplishment and avoid appearing foolish or incapable. Because of their motivation to avoid negative external evaluation, shy individuals are often reluctant to ask teachers for help upon encountering difficulties in their schoolwork. Instead, such individuals often choose to solve difficult problems independently or even to leave problems unsolved. Over time, unsolved problems accumulate and affect shy individuals’ learning adjustment. Nicholls (1984) indicated that with respect to task completion, individuals exhibit self-attention tendency and task attention tendency. Self-attention tendency refers to the focus on improving personal ability, whereas task attention tendency refers to the focus on task completion. Differences in motivational tendencies lead to differences in individual academic help-seeking behavior. These findings suggested that educators can pay more attention to shy students and their manifestations and the cause of learning maladjustment in education, and provide psychological counseling intervention for students’ shyness thus keeping shyness within a reasonable range and minimizing the direct impact of shyness on learning adjustment. For instance, educators can provide positive information about shy students’ performance and achievements, and programs specifically designed to promote shy students’ confidence in their abilities rather than avoiding mistakes. Importantly, educators can encourage shy students to overcome the timidity and restrictions that kept them from others and ask others (teachers, classmates, etc.) for help as one of the ways when they faced problems. After solving the problems successfully, students may build their academic efficacy and be motivated to ask for help next time, so as to help them adapt to their studies.

It is worth noting that the goal orientation that characterizes most shy individuals is not completely negative. Shy individuals who strive to achieve mastery-avoid goals tend to ask classmates for help rather than seeking answers independently or avoiding asking for help. Such individuals strive to improve their abilities and successfully complete tasks to avoid the perception of appearing foolish. Because of these behavioral patterns, shyness does not consistently serve as a negative predictor of students’ learning adjustment. According to self-determination theory, humans are dynamic organisms with innate potential for psychological growth and development (Deci and Ryan, 2000). Individuals engage in behaviors that are beneficial to their own development and based on self-assessments of personal needs and environmental information.

### Limitations and Future Study

This study investigated the relation between shyness and learning adjustment and the potential mechanisms in high school students and found that shyness not only predicted learning maladjustment but indirectly predicted learning maladjustment through the multiple mediating roles of goal orientation and academic help seeking. These findings extend those reported in other studies that have examined the potential effects of shyness on learning adjustment. What’s more, this study extended insights from previous studies on learning adjustment by affirming the multiple mediating role of motivation (goal orientation) and behavior strategies (academic help seeking) in the relation between shyness and learning adjustment among high school students. Furthermore, these findings provided a useful reference point for subsequent intervention on learning adjustment of shy high school students.

This study is certainly not without limitations. First, no causal relationships can be drawn due to the cross-sectional design of this study, future experimental studies are expected to be used to replicate this study. Second, this study focuses on the impact of shyness as an individual variable on learning adjustment of high school students, how the environment variable shaped students’ shyness has been overlooked. For instance, how the examination-oriented education from kindergarten through high school in China shaped students’ shyness, even their learning adjustment, remains unknown. Future longitudinal studies are expected to extend this study. Third, as far as this study is concerned, the results of this study should be generalized only to the background of oriental culture, whether there are some unique findings differentiating from this study under other culture background need to be further explored. Finally, this study only examined the effect of shyness on learning adjustment and its internal mechanism. However, there may be a more complex two-way association between shyness and learning adjustment, that is, shyness not only affects individuals’ learning adjustment. Individual learning adjustment may also affect subsequent level of shyness, which should be investigated in further studies.

## Author Contributions

LL contributed to writing, data analysis, and the design of the work. XW conducted experiments. YC, YL, and FG contributed in polishing the manuscript.

## Conflict of Interest Statement

The authors declare that the research was conducted in the absence of any commercial or financial relationships that could be construed as a potential conflict of interest.

## APPENDIX

**Table A1:** Correlations among variables (*n* = 677).

	1	2	3	4	5	6	7	8	9	10	11	12	13	14	15	16	17	18	19	20
1	1																			
2	0.84**	1																		
3	0.86**	0.66**	1																	
4	0.67**	0.44**	0.43**	1																
5	0.82**	0.56**	0.69**	0.42**	1															
6	0.47**	0.38**	0.31**	0.14**	0.23**	1														
7	-0.19**	-0.30**	-0.18**	-0.02	-0.11**	-0.05	1													
8	0.05	-0.10	-0.01	0.38**	0.04	-0.20**	0.37**	1												
9	0.16**	0.04	0.06	0.36**	0.08*	0.03	0.34**	0.45**	1											
10	0.43**	0.37**	0.33**	0.50**	0.33**	-0.03	-0.01	0.38**	0.34**	1										
11	-0.18**	-0.24**	-0.15**	-0.08*	-0.14**	-0.01	0.32**	0.10*	0.09*	-0.08*	1									
12	0.06	-0.01	0.05	-0.08*	-0.14**	-0.01	0.06	0.08*	0.15**	0.04	0.25**	1								
13	0.11**	0.15**	0.13**	0.11**	0.01	0.13**	-0.26**	-0.05	-0.11**	0.13**	-0.20**	-0.07	1							
14	0.25**	0.31**	0.21**	0.07	0.09*	-0.09*	-0.32**	-0.05	-0.10**	0.18**	-0.49**	-0.32**	0.46**	1						
15	-0.26**	-0.34**	-0.22**	0.16	0.19	-0.01	0.57**	0.21**	0.22**	-0.07	0.34**	0.16**	-0.23**	-0.34**	1					
16	-0.21**	-0.26**	-0.18**	-0.09*	-0.19**	0.01	0.42**	0.13**	0.20**	-0.09*	0.29**	0.12**	-0.24**	-0.30**	0.75**	1				
17	-0.22**	-0.21**	-0.19**	-0.11**	-0.19**	-0.07	0.24**	0.03	0.06	-0.06	0.16**	0.10**	-0.05	-0.16**	0.49**	0.20**	1			
18	0.03	-0.07	-0.01	0.21**	0.01	-0.05	0.19**	0.41**	0.29**	0.20**	0.09*	0.12**	0.03	-0.06	0.45**	0.18**	0.08*	1		
19	-0.16**	-0.20**	-0.10*	-0.15**	-0.11**	0.00	0.38**	-0.02	0.01	-0.14**	0.17**	0.02	-0.16**	-0.19**	0.64**	0.34**	0.17**	0.08*	1	
20	-0.24**	-0.29**	-0.20**	-0.13**	-0.16**	-0.04	0.46**	0.12**	0.13**	-0.09*	0.28**	0.13**	-0.23**	-0.28**	0.68**	0.45**	0.18**	0.08*	0.29**	1
M	2.89	2.59	2.55	3.32	2.54	3.65	3.19	3.33	3.54	2.36	3.14	4.01	1.72	2.30	0.65	0.65	0.58	0.77	0.55	0.69
SD	0.63	0.80	0.92	0.87	0.87	0.64	0.67	0.80	0.80	0.82	0.85	0.74	0.61	0.61	0.14	0.25	0.22	0.21	0.24	0.20

*1, shyness; 2, self-expression shyness; 3, shyness toward novelty; 4, shyness for negative social evaluation; 5, shyness toward the opposite sex; 6, unassuming shyness; 7, mastery-approach; 8, performance-approach; 9, mastery-avoid; 10, performance-avoid; 11, instrumental help seeking from teachers; 12, instrumental help seeking from classmates; 13, executive help seeking; 14, avoidance of help seeking; 15, learning adjustment; 16, learning habits; 17, utilization of learning resources; 18, learning motivations; 19, learning satisfactions; 20, learning styles. ^∗^*p* < 0.05, ^∗∗^*p* < 0.01, ^∗∗∗^*p* < 0.001.*

**Table A2:** Model fit indices.

Model	χ^2^/*df*	RMSEA	SRMR	GFI	AGFI	CFI	TLI
Model 1	4.345	0.070	0.056	0.960	0.933	0.931	0.906
Model 2	3.548	0.061	0.051	0.950	0.908	0.932	0.889
Model 3	3.029	0.052	0.036	0.965	0.936	0.943	0.922
Model 4	3.237	0.058	0.049	0.946	0.916	0.930	0.912

**Table A3:** Bias-corrected bootstrap test on mediating effects.

Independent variable (shyness)	Effects	95% bootstrap CI	
		
		Low	High
→ Learning adjustment	-0.0300***	-0.0431	-0.0170
→ Mastery approach → learning adjustment	0.0193***	-0.0287	-0.0106
→ Instrumental help-seeking(teacher) → learning adjustment	-0.0057***	-0.0104	-0.0024
→ Mastery approach → avoidance of help seeking	0.0076***	0.0040	0.0123
→ Mastery avoid → avoidance of help seeking	-0.0019*	-0.0050	-0.0001
→ Performance avoid → avoidance of help seeking	0.0089***	0.0028	0.0155
→ Mastery approach → instrumental help seeking(teacher) → learning adjustment	-0.0018*	-0.0035	-0.0009
→ Mastery approach → executive help seeking → learning adjustment	-0.0007*	-0.0018	-0.0001
→ Mastery avoid → instrumental help seeking(classmate) → learning adjustment	0.0006*	0.0002	0.0014
→ Mastery avoid → executive help seeking → learning adjustment	0.0007*	0.0002	0.0018
→ Performance avoid → executive help seeking → learning adjustment	-0.0019*	-0.0041	-0.0002

*^∗^*p* < 0.05, ^∗∗^*p* < 0.01, ^∗∗∗^*p* < 0.001.*
